# Standardized Pre- and Early Postoperative Dietary Protocols After Bariatric and Metabolic Surgery: A 20-Year Single-Center Experience Across Roux-en-Y Gastric Bypass, Sleeve Gastrectomy, and Ileal Procedures

**DOI:** 10.1007/s11695-026-08786-z

**Published:** 2026-06-23

**Authors:** Magda Rosa Ramos da Cruz, Alcides José Branco Filho, Felipe Ramos Quadros, Maria Vithoria Cordeiro Arruda, Marcos Leal Brioschi

**Affiliations:** 1 Pontifical Catholic University of Paraná – PUCPR, Curitiba, Brazil; 2 Alcides Branco Medical Associates Clinic, Curitiba, Brazil; 3 Alcides Branco Medical Associates Clinic, Curitiba, Brazil; 4 University of São Paulo - USP, Sao Paulo, Brazil; 5 Harvard TH Chan Public Health, MA, USA

**Keywords:** Bariatric surgery, Gastric bypass, Gastrectomy, sleeve, Diet therapy, Postoperative care, Gastrointestinal symptoms, Nutritional status, Retrospective studies

## Abstract

**Background:**

Structured dietary management before and after bariatric and metabolic surgery is essential for reducing early postoperative complications, yet evidence describing tolerance and clinical outcomes across different surgical techniques remains limited. This study evaluated a standardized preoperative preparation diet and a uniform postoperative consistency-progression protocol implemented over two decades in a high-volume reference center.

**Methods:**

This retrospective cohort included 622 adult patients who underwent Roux-en-Y gastric bypass (RYGB), sleeve gastrectomy (SG), ileal interposition, SADI-S, or gastric bipartition between 2004 and 2024. All patients completed a 10-day anti-inflammatory preoperative diet followed by a two-stage liquid regimen. Postoperatively, patients followed a structured progression from liquid to pureed and finally to solid foods over 30 days. Early outcomes included gastrointestinal symptoms and the occurrence of fistulas or drain use.

**Results:**

The cohort comprised 66.9% women, with RYGB being the most frequent procedure (55.5%), followed by SG (24.4%), ileal interposition (14.0%), SADI-S (3.9%), and bipartition (2.3%). Overall prevalence in the first postoperative month was 9.0% for constipation, 2.7% for diarrhea, 8.8% for vomiting, 2.4% for nausea, 0.6% for reflux, and 0.2% for dumping. Fistulas and drain requirements each occurred in 0.5% of patients. Across techniques, 79.9% of patients remained symptom-free; ileal interposition showed the highest symptom rate (29.9%).

**Conclusions:**

A uniform progression protocol following a structured preoperative diet was associated with low rates of gastrointestinal symptoms and postoperative complications across multiple bariatric procedures. These findings suggest that a physiology-based dietary pathway may be safely implemented during early postoperative recovery. Larger prospective studies are warranted, particularly for emerging ileal techniques.

## Introduction

 Obesity is a global epidemic associated with reduced quality of life and a broad spectrum of comorbidities, including type 2 diabetes, hypertension, and cardiovascular disease [1–3]. Bariatric and metabolic surgery (BMS) remains one of the most effective interventions for patients with severe obesity, with demonstrated benefits in sustained weight loss and remission of obesity-related diseases [4]. Over the decades, the field has evolved from early malabsorptive operations to refined procedures such as Roux-en-Y gastric bypass (RYGB), sleeve gastrectomy (SG), and more recent ileal-based techniques including ileal interposition, SADI-S, and gastric bipartition [5–10].

Although the surgical component is central, perioperative nutritional management is equally critical for optimizing safety, promoting recovery, and initiating long-term dietary adaptation. The immediate postoperative period is particularly sensitive, as patients must adhere to staged progression of food consistencies while maintaining adequate hydration and protein intake. Evidence suggests that structured preoperative diets can reduce liver volume, improve metabolic status, and facilitate safer surgical manipulation [11–13]. Similarly, postoperative dietary progression aims to minimize mechanical stress on the anastomoses, support healing, and prevent complications such as vomiting, reflux, diarrhea, and nutritional intolerance.

Despite the recognized importance of diet therapy, there is considerable variability among centers regarding the sequence, duration, and composition of pre- and postoperative dietary protocols. Recent publications highlight the lack of consensus and the limited data on tolerance, symptom patterns, and complication rates across different bariatric procedures [14–16]. As innovative techniques such as ileal interposition and SADI-S become more widely adopted, understanding the interplay between altered gut physiology and early dietary tolerance becomes increasingly relevant.

For more than 20 years, our high-volume specialty center has applied a standardized preoperative diet and a staged postoperative progression protocol for all bariatric and ileal procedures. Initial data from RYGB patients published in 2004 demonstrated promising outcomes [11]; however, no comprehensive evaluation has been performed since these protocols were expanded and adapted to newer surgical techniques.

This study aims to describe the early clinical outcomes associated with the implementation of this standardized dietary pathway, focusing on gastrointestinal symptoms and complications within the first 30 postoperative days across five distinct bariatric and ileal procedures.

## Methods

- Study Design and Setting

This was a retrospective cohort study conducted at a high-volume bariatric and metabolic surgery reference center. The center has applied a standardized preoperative preparation diet and a uniform postoperative dietary progression protocol since 2002. Data were extracted from the electronic medical record system and anonymized using numeric codes to ensure confidentiality.

b. Participants

The initial dataset included 1,000 adults who underwent bariatric or ileal metabolic procedures between January 2004 and December 2024. Patients were eligible if they were aged 18–65 years, completed the structured preoperative nutritional program, and adhered to the postoperative dietary protocol. This study was approved by the Institutional Review Board/Ethics Committee of Pontifical Catholic University of Paraná under protocol number 7.267.820, in accordance with the Declaration of Helsinki. Due to the retrospective nature of the study and anonymized data collection, the requirement for informed consent was waived. The study aimed to evaluate outcomes associated with the standardized protocol and the adherence to the dietary protocol was routinely assessed during scheduled postoperative nutritional follow-up visits. Patients who failed to complete the standardized nutritional follow-up or did not adhere to the dietary protocol were excluded from the final analysis to reduce heterogeneity in postoperative dietary exposure. After exclusions, **622 patients** comprised the analytical cohort.

c. Dietary Protocol

## Preoperative Diet

Ten days before surgery, patients followed an anti-inflammatory diet emphasizing whole, minimally processed foods and excluding ultra-processed items, fried foods, refined sugars, carbonated beverages, and alcohol. Two days before surgery - eight days after starting the anti-inflammatory diet, patients transitioned to a liquid–pureed diet to reduce intestinal residue. On the day preceding surgery, a restricted liquid, residue-free diet was prescribed to optimize gastrointestinal clearance. A refined, low-residue diet, as this protocol includes during the last 2 days of the 10-day preparation diet, is not necessarily anti-inflammatory because of the low fiber content, but rather aims to remove residue from the intestines in preparation for the surgical procedure.

## Postoperative Diet Progression

A unified consistency-progression protocol was applied across all procedures—RYGB, SG, ileal interposition, SADI-S, and gastric bipartition—with adjustments only for gastric volume limits.

The sequence consisted of:

- Days 1–10: liquid diet.
- Days 11–12: liquid-pureed diet.
- Days 13–20: pureed diet.
- From Day 21: soft-solid diet, while avoiding added sugars, high fat foods, alcohol, and carbonated beverages.

Protein intake targets followed established guidelines, including the Enhanced Recovery After Surgery (ERAS) recommendations, aiming for 56 g/day for men and 46 g/day for women [12], or 20–60 g/day [13]. Detailed volume limits by procedure are presented in Table 1.

**Table 1:** Standardized postoperative diet progression according to surgical technique

Diet phase	RYGB (Days 1–10)	Days 11–12	Days 13–20	Days 21–30
Consistency	Liquid	Liquid–pureed	Pureed	Soft-solid
Volume per feeding	30–50 mL	50–70 mL	70–100 mL (≈ 2 tbsp)	100–150 mL (≈ 3 tbsp)
Frequency	Every 2 h	Every 2 h	Every 2 h	Every 2 h
Straining/Texture	Strained through fine sieve	Not strained; blended	Not blended; uniform texture	Whole foods; no raw fruits/vegetables, seeds, or peels
Hydration	≥ 2 L/day	≥ 2 L/day	≥ 2 L/day	≥ 2 L/day
Protein intake	10–15 g, 4×/day	10–15 g, 4×/day	10–15 g, 5×/day	10–15 g, 5×/day
Diet Phase	**SG**,** II**,** SADI-S**,** Bipartition)**	**Days 11–12**	**Days 13–20**	**Days 21–30**
Consistency	Liquid	Liquid–pureed	Pureed	Soft-solid
Volume per feeding	100 mL	100–150 mL	150 mL (≈ 3 tbsp)	150–200 mL (≈ 3 tbsp)
Frequency	Every 2 h	Every 2 h	Every 2 h	Every 2 h
Straining/Texture	Strained through fine sieve	Not strained; blended	Not blended; uniform texture	Whole foods; no raw fruits/vegetables, seeds, or peels
Hydration	≥ 2 L/day	≥ 2 L/day	≥ 2 L/day	≥ 2 L/day
Protein intake	10–15 g, 4×/day	10–15 g, 4×/day	10–15 g, 5×/day	10–15 g, 5×/day

Table Notes:

*• BMI* body mass index, *RYGB* Roux-en-Y gastric bypass, *SADI-S* single anastomosis duodeno-ileal bypass with sleeve, *SG* sleeve gastrectomy

In addition to the standardized dietary protocol, the main bariatric and metabolic procedures routinely performed by the surgical team at our center include sleeve gastrectomy, intestinal bipartition, SADI-S (single anastomosis duodeno-ileal bypass with sleeve), Roux-en-Y gastric bypass, and ileal interposition. Sleeve gastrectomy consists of the longitudinal resection of approximately 70–85% of the stomach, creating a tubular gastric reservoir while preserving the pylorus and physiological intestinal transit. Intestinal bipartition combines sleeve gastrectomy with a distal gastroileal anastomosis, promoting dual alimentary transit with a standardized common channel of approximately 250–300 cm. SADI-S combines sleeve gastrectomy with a single anastomosis between the proximal duodenum and distal ileum, preserving pyloric function and maintaining an approximately 250–300 cm common channel. Roux-en-Y gastric bypass is performed through the creation of a gastric pouch with an estimated volume of 30–50 mL, associated with Roux-en-Y intestinal reconstruction using alimentary and biliopancreatic limbs measuring approximately 120 cm each. Finally, ileal interposition consists of the proximal transposition of a distal ileal segment ranging from 100 to 170 cm, usually to the duodenal region with an excluded bipancreatic loop of approximately 50 cm, aiming to enhance the incretin response and metabolic control.

d. Outcomes

## Primary Outcomes

Early postoperative complications within 30 days, including:

- gastrointestinal symptoms (vomiting, nausea, diarrhea, constipation, reflux, dumping).
- fistula occurrence.
- intraoperative drain placement (as a marker of technical complexity).

e. Data Collection

Demographic and clinical variables (age, sex, weight, height, BMI, surgical technique) were extracted from electronic records. Gastrointestinal symptoms were recorded by the clinical team during routine follow-up visits or documented patient reports. All data were transferred to a secure spreadsheet for analysis.

f. Statistical Analysis

Descriptive statistics were used to summarize demographic variables, symptom frequencies, and complication rates. Categorical variables were presented as counts and percentages. Given the descriptive nature and heterogeneous sample sizes across surgical techniques, no inferential statistical testing was performed.

g. Ethics

The study used anonymized retrospective data and complied with national regulations for research involving human health information. To preserve confidentiality, no identifiable patient data, imaging, or document copies were retained.

## Results

A total of 622 patients met eligibility criteria and completed the standardized pre- and postoperative dietary protocols between 2004 and 2024. Of these, 66.9% (*n* = 416) were women and 33.1% (*n* = 206) were men. The most common procedure was Roux-en-Y gastric bypass (RYGB), performed in 55.47% of patients (*n* = 345), followed by sleeve gastrectomy (SG) (24.44%, *n* = 152), ileal interposition (II) (13.99%, *n* = 87), SADI-S (3.86%, *n* = 24), and gastric bipartition (2.25%, *n* = 14) (Table 2).

**Table 2:** Preoperative demographic characteristics by surgical technique

Variable	Gastric bipartition (*n* = 14)	RYGB (*n* = 345)	SG (*n* = 152)	II (*n* = 87)	SADI-S (*n* = 24)	Total (*n* = 622)
Female, n (%)	4 (28.57%)	287 (83.19%)	85 (55.92%)	31 (35.63%)	9 (37.5%)	416 (66.88%)
Male, n (%)	10 (71.43%)	58 (16.81%)	67 (44.08%)	56 (64.37%)	15 (62.5%)	206 (33.12%)
Age, years (mean ± SD)	43.79 ± 11.70	39.60 ± 10.09	37.33 ± 12.26	46.55 ± 11.73	50.21 ± 14.10	40.52 ± 11.58
Weight, kg (mean ± SD)	128.43 ± 17.43	107.74 ± 20.15	109.76 ± 23.80	110.06 ± 19.16	107.93 ± 18.92	109.03 ± 21.04
Height, m (mean ± SD)	1.76 ± 0.10	1.65 ± 0.09	1.70 ± 0.11	1.72 ± 0.09	1.68 ± 0.09	1.67 ± 0.10
BMI, kg/m² (mean ± SD)	41.45 ± 5.88	39.48 ± 5.14	37.71 ± 5.21	36.90 ± 4.89	37.95 ± 4.74	38.67 ± 5.23

Table Notes:

• Values are reported as mean ± SD

• *BMI* body mass index, *RYGB* Roux-en-Y gastric bypass, *SADI-S* single anastomosis duodeno-ileal bypass with sleeve, *SG* sleeve gastrectomy

Across all techniques, 79.9% of patients reported no gastrointestinal symptoms during the first postoperative month. Among symptomatic patients, 16.7% reported a single symptom, 2.41% reported two symptoms, and 0.96% presented with three or more symptoms.

Table 3 summarizes symptom rates by procedure. Constipation was the most frequent symptom (9.0%, *n* = 56), followed by vomiting (8.8%, *n* = 55), diarrhea (2.7%, *n* = 17), nausea (2.4%, *n* = 15), reflux (0.6%, *n* = 4), and dumping syndrome (0.2%, *n* = 1). Ileal interposition had the highest combined symptom prevalence (29.88%), followed by RYGB (28.70%).

Regarding major complications, the overall prevalence of digestive fistulas was 0.48% (*n* = 3), and drain placement—used as an indicator of intraoperative technical difficulty—also occurred in 0.48% (*n* = 3). All patients who developed fistulas had undergone placement of a surgical drain intraoperatively.

**Table 3:** Early postoperative complications and gastrointestinal symptoms according to surgical technique

Variable	Gastric bipartition (*n* = 14)	RYGB (*n* = 345)	SG (*n* = 152)	II (*n* = 87)	SADI-S (*n* = 24)	Total (*n* = 622)
Leak	0 (0%)	2 (0.6%)	1 (0.7%)	0 (0%)	0 (0%)	3 (0.5%)
Drain use	0 (0%)	2 (0.6%)	1 (0.7%)	0 (0%)	0 (0%)	3 (0.5%)
Dumping	0 (0%)	1 (0.3%)	0 (0%)	0 (0%)	0 (0%)	1 (0.2%)
Constipation	0 (0%)	40 (11.6%)	6 (3.9%)	8 (9.2%)	2 (8.3%)	56 (9.0%)
Diarrhea	0 (0%)	7 (2.0%)	1 (0.7%)	8 (9.2%)	1 (4.2%)	17 (2.7%)
Vomiting	1 (7.1%)	37 (10.7%)	5 (3.3%)	8 (9.2%)	4 (16.7%)	55 (8.8%)
Reflux	0 (0%)	1 (0.3%)	1 (0.7%)	0 (0%)	2 (8.3%)	4 (0.6%)
Nausea	2 (14.3%)	9 (2.6%)	1 (0.7%)	2 (2.3%)	1 (4.2%)	15 (2.4%)

Table Notes:

• Values are reported as *n (%)*

• Symptoms and complications were assessed during the first 30 postoperative days

The distribution of symptoms and complications by surgical technique is presented in Table 3.

## Discussion

This study evaluated the early clinical outcomes of a standardized preoperative diet and postoperative consistency-progression protocol applied uniformly across five bariatric and ileal metabolic procedures. The overall prevalence of gastrointestinal symptoms and major complications in the first postoperative month was low, suggesting that a structured nutritional pathway may contribute to safe recovery during the most vulnerable phase after surgery.

Constipation was the most frequent symptom in our cohort, although its prevalence remained lower than that reported in previous studies, where rates of up to 27% have been described following RYGB after six months [17]. Early postoperative constipation is expected due to reduced dietary fiber, decreased oral intake, and transient motility changes. Female sex, which predominated in our sample, is also a known risk factor. The slightly higher rates observed after ileal metabolic procedures may relate to physiological mechanisms such as the “ileal brake,” in which L-cell secretion of GLP-1 and peptide YY slows gastric emptying and small-bowel transit [18].

The rate of digestive fistula in this study (0.48%) was notably lower than that reported in long-term cohorts, which describe rates between 1.18% and 6% [19, 20]. All fistulas occurred in patients who had undergone intraoperative drain placement, a surrogate marker of technical difficulty, suggesting that patient-related and technical factors may have played a greater role than the dietary protocol itself. Nevertheless, the very low prevalence of this major complication supports the safety of the nutritional pathway during the critical healing period. For emerging ileal procedures, ongoing evaluation with larger and more homogeneous samples is warranted.

Nausea and vomiting are commonly reported after bariatric surgery, with some studies noting rates between 20 and 70% in the first month [21, 22]. In contrast, the prevalence observed in our cohort was lower. While SG has been traditionally associated with higher rates of nausea and vomiting compared with RYGB [21], our findings showed no such pattern, likely influenced by the smaller number of SG patients relative to RYGB. Procedures involving exclusion of the duodenum and proximal jejunum—such as SADI-S, ileal interposition, and gastric bipartition—may predispose patients to nausea via enhanced ileal brake activity and delayed gastric emptying [23]. The higher symptom burden seen in ileal interposition and SADI-S patients aligns with this physiological rationale.

Diarrhea rates were also lower than those typically reported in the literature, where prevalence may reach 10–20% at six months [23]. Early postoperative dietary restrictions—particularly reduced fat intake—may partially explain the lower prevalence observed in the first 30 days. The higher occurrence of diarrhea after ileal interposition further highlights the need for refined dietary progression strategies tailored to ileal-based metabolic procedures.

Reflux was infrequent overall and occurred mainly in SADI-S patients, consistent with the presence of a sleeve component in this procedure. Increased intragastric pressure following sleeve creation has been identified as a contributor to postoperative reflux [24, 25]. The low reflux rate among SG patients in this cohort may reflect conservative portion volumes and careful texture progression during the early postoperative period.

Dumping syndrome was rare, with only one case after RYGB, contrasting with the previously reported prevalence of over 44% in patients with type 2 diabetes compared with 5.6% in the control group [26] and approximately 12% in patients who were assessed 1 or 2 years after surgery using the Dumping Symptom Rating Scale (DSRS) [27]. Restricted intake of simple sugars and gradual consistency progression likely attenuated rapid gastric emptying, supporting the role of nutritional guidance in reducing this complication in our population.

Taken together, these findings underscore that a structured, physiology-informed dietary pathway may be associated with lower rates of early symptoms and complications across diverse bariatric procedures. The protocol’s uniformity across techniques simplifies dietary counseling while maintaining safety; however, emerging ileal operations may require future adaptations to optimize tolerance. As surgical innovation continues to evolve, the interaction between altered gastrointestinal physiology and postoperative dietary progression warrants further investigation. Overall, these results complement current perioperative nutrition guidelines by providing real-world evidence supporting the safety and tolerability of a standardized, staged diet progression in the early postoperative period.

## Limitations

This study has several limitations. Its retrospective design limits control over confounding factors and restricts causal interpretation. Sample sizes were unbalanced across surgical techniques, particularly for ileal procedures, which may reduce the precision of symptom estimates and limit generalizability. Gastrointestinal symptoms were based on patient reports obtained during routine follow-up rather than on validated symptom scores, which may introduce reporting bias and the preoperative gastrointestinal symptoms were not systematically available. In addition, the scarce literature reporting alternative dietary protocols and their associated early postoperative complications made it difficult to contextualize the relative effectiveness of our protocol.

## Conclusion

In this large retrospective cohort, implementation of a structured preoperative preparation diet and a staged postoperative progression protocol was associated with low early complication rates and high overall dietary tolerance across multiple bariatric and ileal metabolic procedures. The protocol demonstrated particularly low rates of digestive fistula, vomiting, and dumping syndrome, suggesting that a carefully staged advancement of diet may support safe early recovery. Further prospective studies with balanced sample sizes across surgical techniques, particularly ileal-based metabolic procedures, are required to refine dietary recommendations and validate the safety profile observed in this cohort.

## Data Availability

No datasets were generated or analysed during the current study.
